# Utility of an Algorithm to Increase the Accuracy of Medication History in an Obstetrical Setting

**DOI:** 10.1371/journal.pone.0151205

**Published:** 2016-03-21

**Authors:** Aline Corbel, David Baud, Aziz Chaouch, Johnny Beney, Chantal Csajka, Alice Panchaud

**Affiliations:** 1 School of Pharmaceutical Sciences, University of Geneva and University of Lausanne, Geneva, Switzerland; 2 Department of Gynecology and Obstetrics, University Hospital of Lausanne, Lausanne, Switzerland; 3 Institute of Social and Preventive Medicine, University Hospital of Lausanne, Lausanne, Switzerland; 4 Division of Pharmacy, Central Institute of the Valais Hospitals, Sion, Switzerland; 5 Division of Clinical Pharmacology, University Hospital of Lausanne, Lausanne, Switzerland; PreTel Inc, UNITED STATES

## Abstract

**Background:**

In an obstetrical setting, inaccurate medication histories at hospital admission may result in failure to identify potentially harmful treatments for patients and/or their fetus(es).

**Methods:**

This prospective study was conducted to assess average concordance rates between (1) a medication list obtained with a one-page structured medication history algorithm developed for the obstetrical setting and (2) the medication list reported in medical records and obtained by open-ended questions based on standard procedures. Both lists were converted into concordance rate using a best possible medication history approach as the reference (information obtained by patients, prescribers and community pharmacists’ interviews).

**Results:**

The algorithm-based method obtained a higher average concordance rate than the standard method, with respectively 90.2% [CI_95%_ 85.8–94.3] *versus* 24.6% [CI_95%_15.3–34.4] concordance rates (p<0.01).

**Conclusion:**

Our algorithm-based method strongly enhanced the accuracy of the medication history in our obstetric population, without using substantial resources. Its implementation is an effective first step to the medication reconciliation process, which has been recognized as a very important component of patients’ drug safety.

## Introduction

A 38-years-old woman, 20 weeks of pregnancy, was admitted to the Prenatal unit at the University Hospital of Lausanne in Switzerland with anhydramnios and intrauterine growth restriction without evidence of ruptured membranes. All investigations (e.g. genetic, infectious) were normal. Termination of pregnancy was discussed but declined by the patient just before a harmful treatment was identified. The patient was using olmesartan, an angiotensin-II receptor 1 antagonist (AT_1_-antagonist), for several years to treat her arterial hypertension despite the clearly established fetotoxic potential of AT_1_-antagonists (i.e. associated with oligohydramnios or anhydramnios, skull and lung hypoplasia and fetal or neonatal death) [1]. The imputability of olmesartan in the onset of anhydramnios was considered as likely in the absence of other etiologies and it was replaced by a beta-blocker compatible with pregnancy. The amniotic fluid restored and the patient delivered at 33 weeks of pregnancy a healthy preterm girl born with no pulmonary or skull hypoplasia.

This case illustrates how, in an obstetrical setting, inaccurate medication history undertaken at hospital admission can result in failure to identify potentially harmful treatments for patients and/or their fetus. The problem of inaccurate medication lists at hospital admission and discharge has gained much attention in recent years, specifically with regard to patient safety [2, 3]. Medication reconciliation has been recommended by patient safety organizations and authorities to reduce the inaccuracy in patient’s medication lists [4]. Medication reconciliation is defined as the formal and standardized process of obtaining the complete list of a patient's previous medications when performing a transition between health centers, comparing it to the current prescription, and analyzing and resolving any discrepancies [5].

Providing an accurate medication history is a crucial first step in the process of medication reconciliation. Errors in the medication history can be classified into omission errors (drugs missed from the history), commission errors (drugs added to the history), frequency errors, and dose errors. Omission errors, as observed in the clinical case described above, have been reported to affect up to 60% of patients at admission [3]. Strategies to obtain the most comprehensive medication history include the best possible medication history. It can be defined as a medication history that includes a thorough history of all regular medication use (prescribed and non-prescribed, complementary or herbal medicines), using a number of different sources of information (e.g. family or caregiver, community pharmacists and physicians, patient medication lists, previous patient health records). This approach is different and more comprehensive than a routine primary medication history (which is often a quick patient medication history) [6]. Traditionally, medication history is obtained by quick and most often open-ended questions due to lack of time and resources. In general, structured questions, involving additional information on drug names and indications for example, are far more complete than are open-ended questions on patients’ recall of medication use. Several studies have shown that a structured routine primary medication history can improve the completeness of the collected information [4, 7]. Furthermore, it has been shown that medication recall is improved when drug name or indications or pictures of drugs are used [8],[9].

As the best possible medication history process requires substantial human resources and is time consuming [10], many organizations endorse the use of medication management information technology (MMIT) to help to perform medication reconciliation. Access information about medication history using multiple electronic data sources or electronic health records (HER) are emerging strategies [11, 12]. The major limitation of the e-approach to create medication history is that many of the listed medications in electronic data sources (up to 70%) are no longer being used by the patient, as medication lists become out-of-date [13]. So, if this strategy can reduce the time to complete the process of gathering information from several sources as required by the best possible medication history approach or reduce the patient’s recall bias [14], it can’t replace the information offered by the patient (or family) through a medication history interview. Furthermore, the use of electronic tools to gather information to create a medication history is only possible in hospitals that have successfully implemented computerized medical record with access to community-based records or HER. Thus, the effective implementation of electronic tools for medication management has proven to be challenging. For example, there have been studies showing a very low rate adoption of optional electronic medication reconciliation applications [15]. Finally, the utility and safety of these electronic approaches has not been formally assessed yet, clinical endpoints are currently subject to further studies [16] [17].

To our knowledge, paper or electronic tools specifically developed to perform accurate medication history in the obstetric population have never been described. Even if a number of studies have found that the use of medication during pregnancy is common [18], the quantity and the type of drug encountered in an obstetric setting differ from other medical area. This study aims to assess the accuracy of the medication history gathered using an algorithm-based method developed for our obstetric population.

## Method

This study enrolled prospectively patients admitted to the prenatal unit between March and May 2014, over the age of 18, with a sufficient understanding of French, after signing an informed consent form.

**Fig 1:**
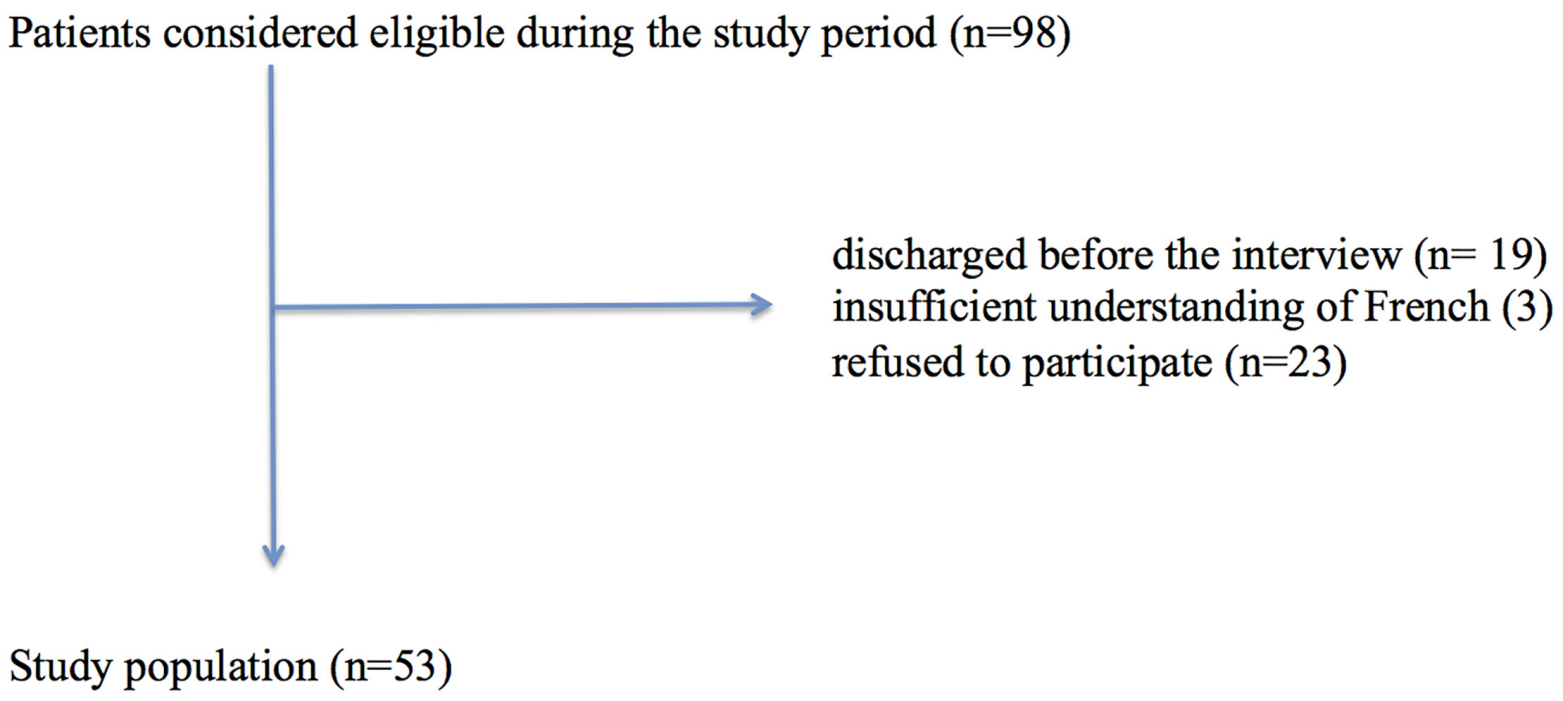
Algorithm dedicated to medication history in an obstetrical setting.

A one-page algorithm (see Fig 1) was developed to allow a structured patient medication interview focusing on the prevention of recall bias. It was elaborated by a multidisciplinary group of healthcare providers (3 midwifes, 1 clinical pharmacist and 2 obstetricians) with expertise in obstetric patient interview, through focus group meetings during a two months period. An algorithm previously developed for internal medicine patients has been used as a starting point [19]. Issues and recommendations specific to the obstetric setting were discussed and used to adapt the previously developed algorithm. An advanced version of the algorithm was tested on a small subset of patients to prevent comprehension issues. This tool was meant to allow a collection of drug information by physicians, pharmacists or midwifes within 10 minutes in an obstetrical setting. The first part of the algorithm contains closed-ended questions targeting current medication, associated side effects and known allergies along with information on names, routes, doses, or frequency of administration. The second part contains pictures representing the different dosage forms (e.g. spray, tablets, injection, eye drops) shown to the patient while readdressing the questions on current medication. The pictures are used as a reminder and allow completing information collected during the first part of the algorithm.

A single investigator conducted the interviews using the algorithm throughout the study. No training was provided to use the tool. The medication history obtained with the algorithm was compared to the list retrieved from the electronic medical record. This list came from open-ended questions asked to the patient by the physician upon admission and represents the standard method for medication history used in the prenatal unit. As reference, the best possible medication history list was gathered by involving patients and interviewing prescribers and community pharmacist whose contact information were collected during the patient interview. Each of the health care professional received a phone call within 24 hours asking them to complete a medication history form requesting for medication names, routes, doses, or frequency of administration. The form was sent by email, mail or fax as preferred. A follow-up call was done after a week if necessary. This best possible approach was considered as the most exhaustive known list of current drugs, as thus assumed to be equal to 100% of the medications taken by the patient. Since the best medication history implicitly contained the list of medications identified with both the algorithm-based and the standard approaches, false positives (i.e. medications identified by either method but not listed in the best medication history method) were not possible by design. Therefore, the analysis was oriented towards the count of true positives and both the algorithm-based and the standard approach-based lists were converted to concordance rates (i.e. proportion of medications correctly identified by each method for a given patient) using the best medication history list as the reference. An independent pharmacist un-blinded to the hypothesis conducted the analysis of concordance.

Differences in average concordance rates between both approaches were determined by the Wilcoxon signed rank test and were considered as statistically significant at p<0.05 (*Stata*, version 13.0, Statacorp, Texas, USA). Confidence intervals for the concordance rates were established using a non parametric bootstrap strategy (*R*, version 3.0.3, Free Software Foundation's).

This work was approved by the Ethics Committee in Research of the University Hospital of Lausanne.

## Results

**Fig 2:**
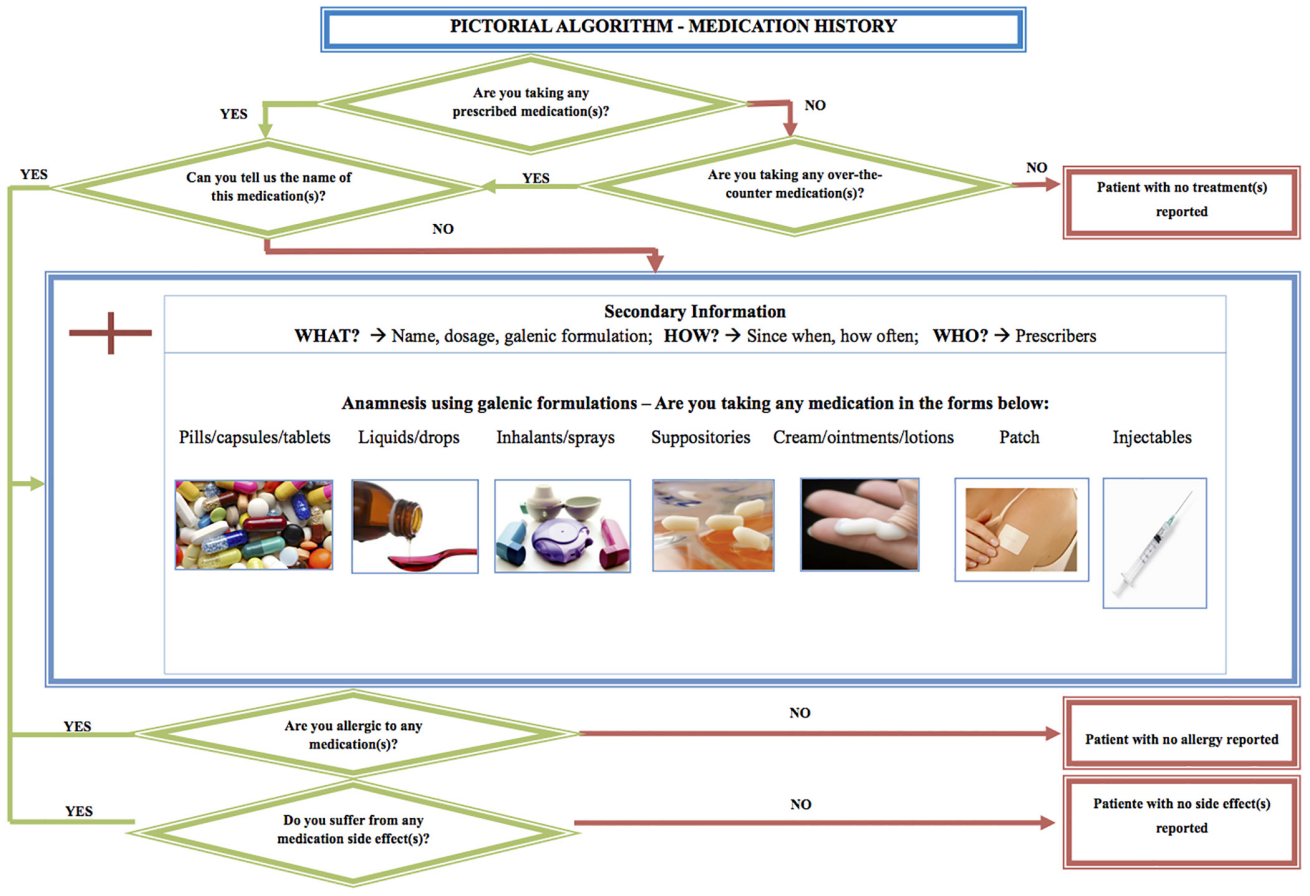
Patient inclusion Flow chart.

A total of 98 patients were considered eligible during the study period. Fifty-three patients (median age [range]: 30 years [range 20–43]; gestational weeks: 29 [range 19–39]) accepted to participate and were interviewed with the help of the algorithm within a mean of 2.6 days [SD ± 1.7 days] after admission (Table 1). The inclusion rate was 55%, 19 were discharged before the interviewed was performed, 3 had insufficient understanding of French and 23 refused to participate (Fig 2).

**Table 1:** Characteristics of Patients.

n = 53	Value	
**Age median (min ; max)**	30	(20;43)
**Marital status (n)**		
**married**	56.6%	(30)
**single**	35.8%	(19)
**divorced**	7.6%	(4)
**French level (n)**		
**≥ sufficient**	98.1%	(52)
**Education level (n)**		
**≥ university**	26.4%	(14)
**Gestational age at admission median (min ; max)**	29	(19;39)
**Gestity median (min ; max)**	2	(1;13)
**Parity median (min ; max)**	0	(0;4)
**Reasons for admission (n)**		
**premature labor**	39.6%	(21)
**metrorrhagia/abnormal bleeding**	18.9%	(10)
**corticosteroids to promote fetal lung maturation**	7.5%	(4)
**oligohydramnios/polyhydramnios**	5.6%	(3)
**pre-eclampsia**	3.8%	(2)
**others**	**24.6**	**(13)**

The median number of medications per patient at admission was 4 [range 0–15]. Removing vitamins, mineral supplements, iron and homeopathic preparations reduced the median number of medications to 1 [range 0–11] with 30% of the treatments used for chronic conditions (e.g. diabetes).

The algorithm-based approach obtained higher average concordance rates than the standard approach, with respectively 90.2% [CI_95%_ 85.8–94.3] vs. 24.6% [CI_95%_ 15.3–34.4] (p<0.01) concordance. Average concordance rates were 91.0% [CI_95%_ 84.9–95.9] vs. 43.8% [CI_95%_ 31.7–57.4] for the algorithm-based and standard approaches, respectively, after removing vitamins, mineral supplements, iron and homeopathic preparations (p<0.01). Higher average concordance rates were also obtained by the algorithm while considering information on routes, doses, or frequency of administration: dosage (64.7% [CI_95%_56.6–72.8] vs. 6.3% [CI_95%_2.2–11.7]), galenic formulations (90.1% [CI_95%_85.7–94.2] vs. 0% [0]) and time of medication intake (89.6% [CI_95%_84.1–94.4] vs. 1% [CI_95%_0–2.9]) for the algorithm and the standard methods, respectively.

At least one drug (excluding vitamins, mineral supplements, iron and homeopathic preparations) was omitted in more than 60% of our 53 patients in the standard approach, compared to 19.6% with the algorithm-based approach. The most omitted drug families were analgesics (number of unidentified (n) = 20 with standard approach vs n = 2 with algorithm-based approach), decongestants and nasal preparations (n = 10 with standard approach vs n = 2 with algorithm-based approach), anti-nausea drugs (n = 6 with standard approach vs n = 1 with algorithm-based approach), hormones (n = 5 with standard approach vs n = 0 with algorithm-based approach), drugs for peptic ulcer and gastroesophageal reflux disease (n = 5 with standard approach vs n = 2 with algorithm-based approach), insulines and analogues (n = 4 with standard approach vs n = 2 with algorithm-based approach), and calcium channel blockers (number of unidentified: n = 4 with standard approach vs n = 2 with algorithm-based approach).

The best medication history list that gathered information from physicians and pharmacists took one week on average to be completed (range 0–5 week). On the other hand, the algorithm-based medication history took less than 10 minutes to be completed. Prescribing physicians and community pharmacies visited within the last 6 months before hospitalization contributed to the best medication history list in 72% of the cases (n = 38). In the remaining 28% of the patients (n = 15), only one of the healthcare professional filled out the medication history form.

## Discussion

This prospective study was conducted to determine the accuracy and evaluate the utility of a one-page structured medication history algorithm in the obstetrical setting. The tested algorithm significantly enhanced the completeness and accuracy of medication history in the tested obstetric sample. To our knowledge, this is the first tool specifically developed to perform accurate medication history in the obstetric population.

According to the Institute for safe medication practices in Canada, the best medication history approach should gather information from as many sources as possible (i.e. patient, physician, pharmacists, family). However, gathering information from community care providers without electronic shared medical records can be time consuming and not always efficient, as observed in our study. This approach significantly expended staff resources as one full time pharmacist was required to accomplish this task. Additionally, it didn’t allow fulfilling the WHO recommendations of formal reconciliation within 24 hours after patient admission because of the time required to get a feedback from physicians and pharmacists [20]. In our setting, the algorithm-based methods offered an optimized approach in the view of completeness of the information obtained, compromise between time and cost investments and compliance with WHO recommendations. This tool could be used in any similar setting using paper or electronic format without significant training. However, the use of this algorithm in other settings (e.g. patients with numerous treatments) could be associated to a lack of completeness.

Unsurprisingly, excluding vitamins, mineral supplements, iron and homeopathic preparations lowered the observed differences between the algorithm-based and standard approach-based average concordance rates. Although these products are sometimes considered as harmless by health providers, they can be associated with side effects or interact with other medication (e.g. iron, magnesium) and they allow gaining a more comprehensive clinical picture of the patient.

Information on routes, doses, or frequency of administration was often missing in the drug lists collected with the standard-based approach. It is likely that these gaps were largely the results of a lack of documentation in the electronic medical record. Incomplete documentation of the medication history has been previously reported as a consequence of lack of time, of physical space in the medical charts or computer space and duplication of work.[21] Nonetheless, documentation of all information is an important step in the process of medication history, as it not only enables the prescription of the proper dosage but also ensures the continuum of care.

In the absence of centralized database storing patients’ medication lists, enhancement of medication recall by the patients using tools, such as pictures, are inexpensive, simple to construct, and easy to implement. In previous studies, pictures have been used in clinical setting with some success.[8] Although not formally evaluated in our study, the level of completeness of the information collected after visualization of the pictures representing the different dosage and pharmaceutical forms (e.g. spray, tablets, injection, eye drops) was increased. The pictures seemed to improve the level of awareness of patients about their medications and suggested that some medications were not recognized as such by the patients (e.g. a cream is not a drug).

This study had several limitations. Firstly, it was conducted in a single hospital and all the interviews have been conducted by a single investigator, which limits the generalizability of the results. Secondly, the best medical history approach was not constructed independently of the two other methods as it did include, among others, all the medications that were identified by both the algorithm and the standard methods. It follows that false positives were impossible by design. Consequently, this study is essentially a sensitivity analysis and does not provide answers regarding the specificity of either method. In order to investigate both the sensitivity and specificity of the two methods, a true gold standard for the medication history should be used but does not currently exists in the absence of electronic shared medical records.

In conclusion, the algorithm-based approach helps standardizing the medication interview and can be easily used by all health care providers (e.g. nurses, physicians or pharmacists). It is an easy, reliable, rapid and cost-sparing method that can be very efficient for the obstetric population in a setting without electronic, shared medical records. Considering the potentially very serious and harmful consequences of inappropriate drug use in pregnancy, such an algorithm will add another level of security in patient care and represents an effective first step to the medication reconciliation process that will help physicians find the most suitable treatment for their patients.
